# From features to dimensions: cognitive and motor development in pop-out search in children and young adults

**DOI:** 10.3389/fpsyg.2014.00519

**Published:** 2014-05-30

**Authors:** Anna Grubert, Marcello Indino, Joseph Krummenacher

**Affiliations:** ^1^Department of Psychological Sciences, Birkbeck College, University of LondonLondon, UK; ^2^Department of Psychology, University of FreiburgFreiburg, Switzerland; ^3^Department of Psychology, University of ZurichZurich, Switzerland; ^4^Neuro-cognitive Psychology, Ludwig-Maximilian-University MunichMunich, Germany

**Keywords:** pop-out, feature-based attention, dimension-based attention, cognitive development, visual search

## Abstract

In an experiment involving a total of 124 participants, divided into eight age groups (6-, 8-, 10-, 12-, 14-, 16-, 18-, and 20-year-olds) the development of the processing components underlying visual search for pop-out targets was tracked. Participants indicated the presence or absence of color or orientation feature singleton targets. Observers also solved a detection task, in which they responded to the onset of search arrays. There were two main results. First, analyses of inter-trial effects revealed differences in the search strategies of the 6-year-old participants compared to older age groups. Participants older than 8 years based target detection on feature-less dimensional salience signals (indicated by cross-trial RT costs in target dimension change relative to repetition trials), the 6-year-olds accessed the target feature to make a target present or absent decision (cross-trial RT costs in target feature change relative to feature repetition trials). The result agrees with predictions derived from the Dimension Weighting account and previous investigations of inter-trial effects in adult observers (Müller et al., 1995; Found and Müller, 1996). The results are also in line with theories of cognitive development suggesting that the ability to abstract specific visual features into feature categories is developed after the age of 7 years. Second, overall search RTs decreased with increasing age in a decelerated fashion. RT differences between consecutive age groups can be explained by sensory-motor maturation up to the age of 10 years (as indicated by RTs in the onset detection task). Expedited RTs in older age groups (10-, vs. 12-year-olds; 14- vs. 16-year-olds), but also in the 6- vs. 8-year-olds, are due to the development of search-related (cognitive) processes. Overall, the results suggest that the level of adult performance in visual search for pop-out targets is achieved by the age of 16.

## Introduction

At any moment in time the visual system is exposed to an abundance of various colors and forms, from which those need to be selected which are conducive to current behavioral goals. One approach to efficient selection is the reduction of visual complexity at an early processing stage by subsuming specific features into feature categories or dimensions. Salience-based models of visual selection (Koch and Ullman, 1985; Wolfe et al., 1989; Cave and Wolfe, 1990; Treisman and Sato, 1990; Wolfe, 1994, 2007; Müller et al., 1995, 2003; Found and Müller, 1996; Itti and Koch, 2000) propose that the allocation of focal attention to a particular location of a visual scene is controlled by a feature-less priority representation (Fecteau and Munoz, 2006). Priority is a space-based activation which is mainly based on bottom-up feature contrast or salience signals and which is susceptible to modulation by a number of factors including inter-trial history and task-set (Müller et al., 1995, 2003; Found and Müller, 1996). Found and Müller (1996) presented their observers with feature targets which differed from distractors (green vertical bars) in the color (red or blue vertical bar) or orientation dimension (green left-tilted or right-tilted bar). Target presence was detected efficiently in a parallel manner. However, mean reaction times (RTs) were systematically modulated by whether the dimension defining the target on the current trial N was repeated or changed relative to the preceding trial N-1. Dimension repetition (trial N-1: color → trial N: color) yielded a relative RT benefit, dimension change (trial N-1: color → trial N: orientation) resulted in a RT cost. Importantly, RTs were statistically unaffected by whether, within a given dimension, the target was defined by the same (e.g., N-1: red → N-red) or by a different feature (e.g., N-1: left-tilted → N: right-tilted). Found and Müller (1996) interpreted their finding as evidence supporting a Dimension Weighting (DW) model of salience-based selection (Müller et al., 1995, 2003; see Krummenacher and Müller, 2012 for a recent review).

According to the DW account, salience signals are generated for a limited number of visual dimensions such as color, orientation, size or motion (see Wolfe and Horowitz, 2004, for an examination of visual dimensions). Dimensions need to be checked serially for the presence of a salience peak by a process that allocates attentional weight (processing resources) to individual dimensions. As attentional weight is limited, processing resources allocated to one dimension need to be de-allocated from other dimensions. Weight which was allocated to one dimension persists (at least) into the next experimental trial, that is, weight allocation is modulated by the recent trial history. For example, if weight was allocated to the color dimension in a color target trial N-1, target detection is expedited if a color target is presented in trial N because processing resources do not need to be shifted to the color dimension. By contrast, if an orientation target is presented in trial N, resources need to be shifted in a time-consuming process to the orientation dimension, giving rise to RT costs relative to inter-trial dimension repetitions. Importantly, attentional weight is allocated to dimensional modules (e.g., color, orientations) rather than specific feature values (e.g., red, blue, left-tilted, right-tilted) as repetition or change of features across trials does not affect search RTs. No feature-based inter-trial effects were observed.

Another important assumption of Müller et al.'s (1995, 2003) DW account is that, in feature search tasks, the detection of the presence of a target does not require that the target's identity is established. Rather, the target present response is based on the presence of a salience peak in one of the dimension-based processing modules.

The mechanism mediating search for feature (pop-out) targets, that is, the generation of dimension-based salience signals from feature representations and the RT benefits and costs associated with shifts of processing resources between dimensions, is well established and understood in adult observers. In contrast, comparably little is known about whether those processes exist in children or at what age they develop.

Theories of development assume that categorization skills are developed during childhood (e.g., Piaget, 1964; Frith and Frith, 1978; Tversky, 1985). With regard to visual search, however, there is only little experimental evidence for the question whether and how child observers categorize visual information, and when during childhood the ability to abstract from visual features is developed. The study by Donnelly et al. (2007) is, to our knowledge, the only one investigating effects of categorizing in children employing a visual search task comparable to the tasks adult observers are usually presented with. Donnelly et al. (2007) examined performance in 6/7-year-old, 9/10-year-old, and adult observers in a task involving search for color (purple, red), orientation (oblique, vertical), and size (small, large) singletons presented within an array of homogeneous distractors. Participants were asked to indicate whether all stimuli in the display looked the same (target absent; e.g., six red vertical bars) or whether one of them differed from all the others (target present, e.g., a red left-tilted bar among five red vertical bars). While 9/10-year-olds and adults detected color and orientation targets equally efficiently, 6/7-year olds were significantly slower on orientation than on color target trials. The authors speculated that participants of the two older age groups were able to simultaneously monitor the visual field for a salient target across multiple feature maps (i.e., they were able to search across dimensions), basing search on bottom-up (salience) signals. By contrast, the 6/7-year-olds searched for the presence of a feature that matched the corresponding template within one of the potential target dimensions (e.g., color) and then switched to the other dimensions (e.g., orientation, size) until a target was detected or all dimensions were checked. Note that the process assumed by Donnelly et al. (2007) is in accordance to the dimension checking mechanism (by weight shifting) proposed by the DW account.

The idea that the ability to categorize objects improves at around the age of 7 years is in line with theoretical accounts of development. For example, in Piaget's (1964) theory of cognitive development children between the age of 2 and 7 years are at the pre-operational stage of cognitive development which is characterized by the inability to mentally manipulate information (children perform physical, but not mental, operations on tasks). In the pre-operational stage the classification of objects relies on specific features only (e.g., all red items are grouped together, regardless of their shape or size). In the subsequent concrete operational phase between the ages of seven and eleven, concrete objects are then appropriately classified according to several features (e.g., all red items are ordered according to their size). Similarly, Frith and Frith (1978) postulated that genuine categorization is achieved between the age of five to eight, but that in that age range children are still only able to attend to one dimension of similarity at a time (e.g., all red items). Only children aged eight truly learn to categorize objects according to multiple levels and to process several visual dimensions of similarity at the same time (e.g., all red, all large, and all round items). Tversky (1985) agrees, but claims that significant cognitive changes associated with the ability to create categorical concepts are related to a child's school enrolment (externally triggered), rather than being the result of a development which is induced at a particular age.

In relation to visual search, the question arises as to whether ignoring irrelevant feature information in favor of what could be referred to as abstracted dimensional (categorical) information is related to the development of these categorization abilities during childhood. The underlying idea of both concepts is that real objects are mentally classified into superordinate constructs that do not exist in the real world. Therefore, it is straightforward to follow that, with no general ability to categorize objects there is no categorization of features, either.

In addition to the effects on search RTs which can be associated with the ability to categorize visual features into dimensions, Donnelly et al. (2007) also reported a main effect of age on RTs: mean search RTs decreased as a function of age. Also, in all age groups, RTs in target absent trials were slower than RTs in target present trials, but the difference between target present and target absent RTs decreased with increasing age. Donnelly et al. concluded that, taken together, the RT differences between the age groups found in their study constitute evidence for the development of adult-like efficiency in visual search between middle to late childhood. Importantly, at least part of the performance improvement is due to the development of cognitive skills related to the reduction (categorization) of visual features into feature categories. The latter conclusion is exciting in that it represents a novel interpretation of improvements of search efficiency as a function of age which had exclusively been attributed to ongoing sensory-motor maturation resulting from the completion of the myelination of sensory and motor pathways (e.g., Trick and Enns, 1998; Gerhardstein et al., 2001; Gerhardstein and Rovee-Collier, 2002; see Adler and Orprecio, 2006, who used the sensory-motor maturation argument to explain longer saccade latencies in infants relative to adults).

The aim of the present study was twofold. First, and most important, we aimed to directly compare the ability to categorize visual features into dimensions in children, adolescents, and young adults in a pop-out search task. Observers aged 6–20 years completed a singleton feature search task while RTs and errors were recorded. For the analysis, participants were categorized into eight age groups (6-, 8-, 10-, 12-, 14-, 16-, 18-, and 20-year-olds). The task was exactly the same for participants of all ages. On target absent trials, all the items of the search display were identical (green vertical bars), on target present trials, one of the distractors was replaced by a red or blue vertical bar (color dimension) or a left-tilted or right-tilted green bar (orientation dimension). Participants were instructed to indicate target presence and absence by pressing one of two predefined response keys. Dimensional and featural target definition was pseudo-randomized across trials in such a way that feature-repetition (e.g., red target preceded by a red target), feature-change (e.g., red target preceded by a blue target), and dimension-change (e.g., red target preceded by a left-tilted target) inter-trial transitions occurred equally often in each block. For each age group RT effects of search across dimensions (RTs on dimension-change compared to feature-change trials; dimension-based inter-trial effects) and across features (RTs on feature-change compared to feature-repetition trials; feature-based inter-trial effects) were investigated.

If, as in Donnelly et al. (2007), and suggested by theories of cognitive development described above, the ability to categorize feature identities into feature dimensions develops at around the age of seven, there should be marked differences in inter-trial effects between the 6-year-olds and all older age groups. If participants aged eight and older are able to search simultaneously and efficiently across multiple feature dimensions, they probably base (feature) target detection on salience signals. Thus, search for feature targets defined on different dimensions would require shifts of processing resources between dimensions. This behavior will generate dimension-based, but not feature-based inter-trial effects across trials as found in studies examining adult observers (Müller et al., 1995, 2003; Found and Müller, 1996). However, if before the age of seven, participants are not able to abstract individual feature identities into categories, participants aged 6 years should not be able to rely on dimensional salience signals. Rather they search for feature targets in a serial fashion, matching single deviant features to memorized target feature templates. This behavior should not lead to dimension-based inter-trial effects. In contrast, within this age group, participants should produce feature-based inter-trial effects. If they classify the target on the basis of single features (because no concept of feature dimensions is yet available), they will establish the exact feature identity of the target on each trial. It is then straight forward to assume that on trial N checking of features is likely to start with the feature that resulted in a match in the previous trial N-1. In other words, in this study different types of inter-trial transitions were used as an operational tool to discriminate between dimension-based (reduced feature-based information) and feature-based (non-reduced visual information) processing of target items (see Krummenacher et al., 2010; Grubert et al., 2013, for similar procedures).

The second aim of the study was to examine overall search RTs as a function of an extended range of ages between 6 and 20 years. In line with the results of previous studies (Trick and Enns, 1998; Gerhardstein et al., 2001; Gerhardstein and Rovee-Collier, 2002; Donnelly et al., 2007) RTs were expected to decrease with increasing age. However, as suggested by Donnelly et al. (2007), if RTs are affected by newly developing cognitive skills (such as the ability to rely target detection responses on the presence of salience peaks rather than the matches between features and templates), RTs, in addition to sensory-motor maturation, are also expected to be modulated by the different cognitive processes invoked to solve the search task in participants aged 6-years relative to participants aged 8 and older. In order to identify such distinct steps in cognitive development search RTs were compared to performance in a simple detection task. In detail, participants were presented with search displays (stimuli were physically identical to the ones presented in the search task) of either homogenous arrays of green vertical bars, or containing a color (red, blue vertical bar) or orientation (green, left-tilted or right-tilted bar) singleton. Participants were instructed to respond as quickly as possible to the onset of the display. They did not decide whether a target was present or not in the display. The simple detection task was introduced in order to isolate pure sensory-motor processing time, which is the time taken to detect the onset of a display and to execute a response. The reasoning is based on Donders' (1969) additive factors logic assuming that RTs in the simple detection task reflect (sensory-motor) processing time of signal detection and response execution stages while the search task involves the additional (cognitive) processes of signal discrimination (target detection) and response selection. The time taken to complete the cognitive processes can thus be obtained by subtracting RTs in the simple detection task (sensory-motor RT) from RTs in the search task. Overall, the procedure allows to separate performance increases that are due to sensory-motor maturation from RT benefits caused by the use of newly developed cognitive skills as a function of increasing age.

## Methods

### Participants

A total of 124 participants took part in the experiment. Observers were divided into eight age groups: 6-year-olds (*N* = 20, mean age = 6, 9 female), 8-year-olds (*N* = 14, mean age = 8, 9 female), 10-year-olds (*N* = 17, mean age = 10, 10 female), 12-year-olds (*N* = 17, mean age = 12, 15 female), 14-year-olds (*N* = 16, mean age = 14, 10 female), 16-year-olds (*N* = 14, mean age = 15.9, 8 female), 18-year-olds (*N* = 12, mean age = 17.9, 5 female), and 20-year-olds (*N* = 14, mean age = 19.3, 7 female). All participants had normal or corrected-to-normal vision, including color vision. All observers were naïve as to the purpose of the experiment and none of them had previous experience in visual search tasks. All participants were pupils from schools in the canton of Zurich, Switzerland, recruited through the Institute of Developmental Psychology of the University of Zurich.

### Stimuli and procedure

**Figure 1:**
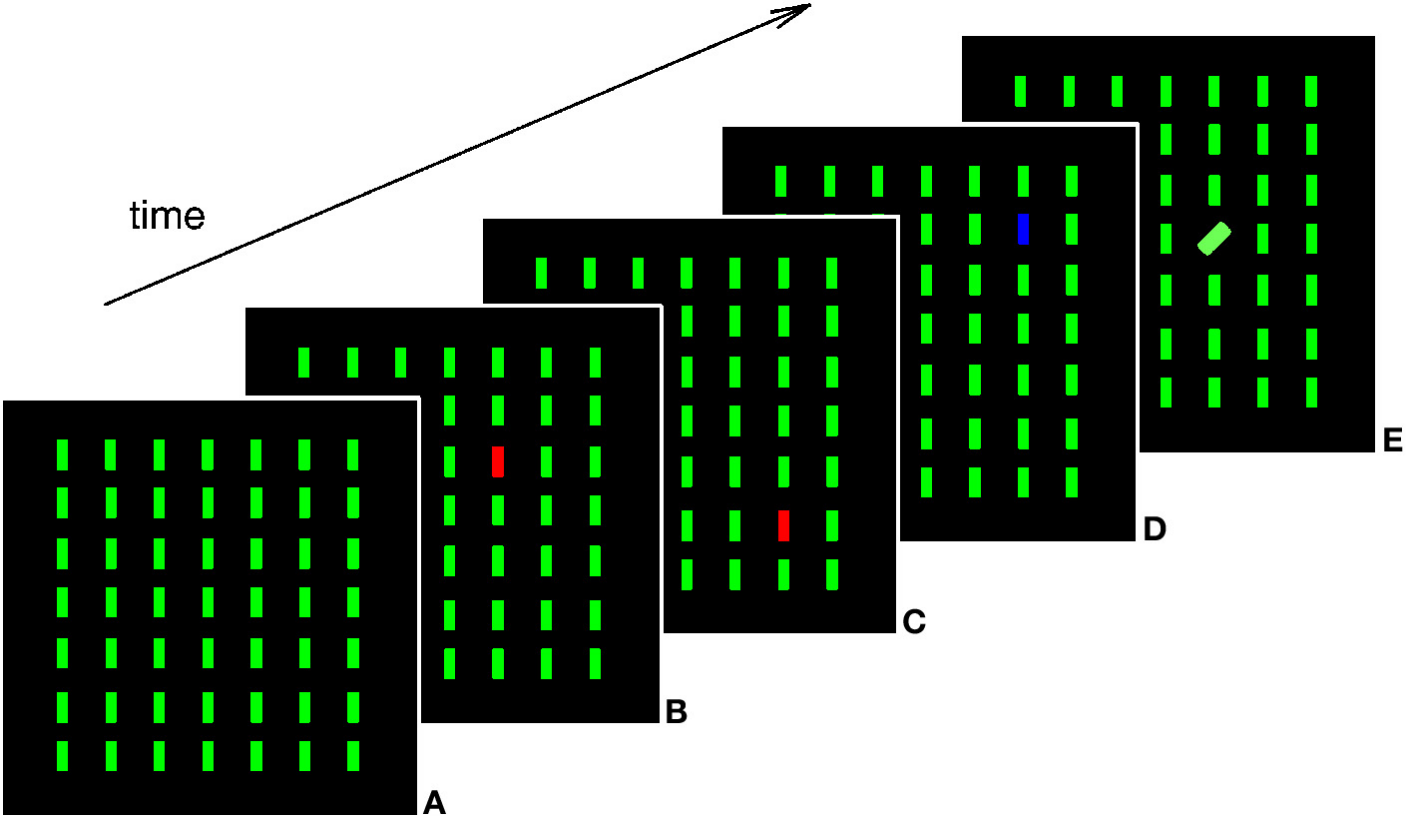
**Schematic illustration of the search displays on target absent (a), target present color (b–d), and orientation (e) trials**. Critical inter-trial transitions between target present trials were feature-repetitions (transition from display b to c), feature-changes within one dimension (transition from display c to d), and dimension-changes (transition from display d to e). Displays were visible until response.

Stimuli were presented on a 15-inch LCD display with a resolution of 1280 × 1024 pixels and a 60 Hz refresh rate. Each participant completed the experiment in an individual session in a dimly lit room made available by the school the participant attended. Observers viewed the display from a distance of approximately 65 cm. Manual responses were registered with the left and right control keys on a standard laptop keyboard. All other keys of the keyboard, with exception of the space bar, were hidden with a custom-made felt cover. Stimulus presentation, timing, and response recording was controlled by an Acer TravelMate 4672LMi laptop running the Windows XP operating system, using the Cogent 2000 toolbox (www.vislab.ucl.ac.uk/Cogent/) for Matlab (Mathworks, Inc.). Non-target stimuli were 49 green (CIE color coordinates: 0.311/0.578) vertically oriented bars (subtending 1° in height and 0.2° in width), arranged within the cells of a virtual grid of 7 rows and 7 columns (with each cell covering an area of the dimensions 1.9° × 1.9° [height × width]) placed at the center of the screen (see Figure 1). Each search item was randomly jittered relative to the center of the cell by a maximum of ±0.5° of visual angle on both the x- and y-axis. On target present trials, one of the non-targets of the central 5 × 5 cells of the virtual grid was replaced by a red (0.596/0.358) or blue (0.148/0.065) vertical bar (color target) or a green bar tilted to the left or right by 45° relative to vertical (orientation target). All search stimuli were equiluminant (at 1.6 cd/m^2^) and presented against a black background. Target locations across trials were randomized. Participants were instructed to report whether all of the display items looked the same (target absent; press the left control key), or whether one of them differed from the others in color or orientation (target present; press the right control key).

Each block comprised 94 trials, 38 of which were target absent, 56 were target present trials. Each of the four target types was presented on 14 trials per block. Inter-trial transitions were pre-programmed and then presented in (pseudo-) random sequences to make sure that each block contained 16 feature-repetition transitions (e.g., red target preceded by a red target), 16 feature-change transitions (e.g., red target preceded by a blue target), and 16 dimension-change transitions (e.g., red target preceded by a left-tilted target), 16 transitions between target absent and present trials (e.g., red target preceded by a target absent trial), and 30 transitions between target absent trials only (e.g., target absent trial preceded by a target absent trial). The four target types were involved equiprobably in each type of inter-trial transition. Participants were familiarized with the task by performing a block of 47 exercise trials before the experiment proper. Following task familiarization, according what was deemed a number of trials appropriate for the age, the 6-year-olds completed three blocks (a total of 282 experimental trials in total), the 8- and 10-year-olds completed four blocks (a total of 376 trials), the 12-year-olds five blocks (a total of 470 trials) and the 14-, 16-, 18-, and 20-year-olds completed six blocks (a total of 564 trials). All participants, irrespective of the number of trials they responded to, took about 30 minutes to complete the experiment. Note that in order to prevent fatigue effects, block length in the experiment was 47 trials and observers were encouraged to take a short break after they had completed a block. Each trial started with the presentation of a white fixation cross, for 700 ms at the center of the screen. Fixation was followed by a blank screen (200 ms) and the onset of the search display, which remained visible until the observer's response. After observers had completed the search experiment, they completed one block of 64 trials of the simple detection task^1^. In the simple detection task, observers were instructed to press the keyboard space bar as quickly as possible after the onset of the stimulus array on the screen. Participants were instructed that the display items they were going to be presented with were the same as in the visual search task they had just completed, but that they would not decide whether all the items were the same or not, rather, they were to press the space bar on the onset of the stimuli and to avoid pressing it before something appeared on the screen. All participants completed a practice block of 32 trials on the detection task to familiarize with the new task instructions.

## Results

Trials with very fast (<200 ms) and very slow (determined by visual inspection of the individual distribution histograms of each age group) responses were excluded from analysis (less than 1.5% of all visual search trials, less than 3.0% of all onset detection trials). In the visual search task, color target trials were responded to faster than orientation target trials in all age groups. However, since in none of the statistical analyses interactions involving target identity were statistically significant, color and orientation trials were pooled. Trials with erroneous responses (false alarms, misses) were excluded from the RT analyses. All *t*-tests are two-tailed and Bonferroni corrected where necessary.

### Reaction times

**Figure 2:**
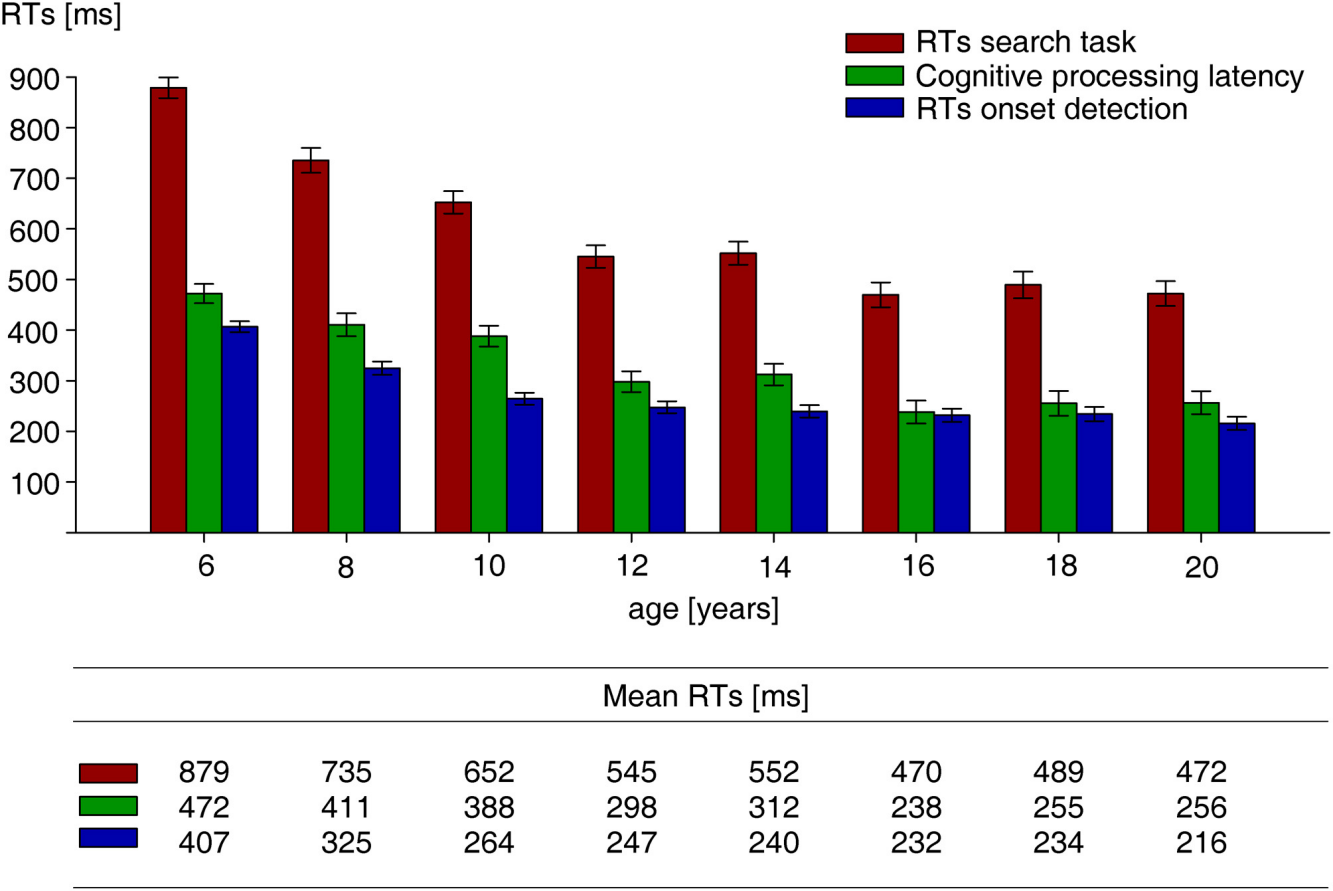
**Mean search RTs on target-present trials of the visual search task, mean RTs of the onset detection task, and mean cognitive processing latencies (obtained by subtracting RTs of the onset detection from RTs of the search task), separately for all age groups**. All RTs are measured in milliseconds. Error bars reflect standard error of the mean.

Figure 2 shows mean RTs on target present trials of the visual search task, mean RTs of the onset detection task (reflecting sensory-motor RTs), and the cognitive processing times obtained by subtracting onset detection RTs from visual search RTs, separately for each age group. Overall, visual search and onset detection RTs decreased with age with the amount of decrease getting smaller with increasing age (decelerated decrease). To identify age groups between which RTs differed significantly, search RTs, detection RTs, and cognitive processing times were subjected to separate univariate analyses of variance (UNIANOVAs) with age as fixed factor. Main effects were followed up by planned repeated contrasts. All three UNIANOVAs revealed significant main effects of age group, all *F*_(7,116)_ > 16.3, all *p* < 0.001. Repeated contrasts (Table 1) showed that RTs to target present trials of the visual search task were significantly faster for the 16- than the 14-year-olds, the 12- than the 10-year-olds, the 10- than the 8-year-olds, and the 8-than the 6-year-olds. More importantly, repeated contrasts on the means of the times taken to complete separate component processes of the search task revealed faster RTs in the onset detection task for the 10- relative to the 8-year-olds, and the 8- relative to the 6-year-olds. Those two differences are suggestive of effects due to sensory-motor maturation. Latency differences between age groups in the (cognitive) processes associated with target identification and response selection (obtained by subtraction RTs in the onset detection task from RTs in the search task) were observed for the 16- relative to the 14-year-olds, the 12- relative to the 10-year-olds, and the 8- relative to the 6-year-olds. Those differences are suggestive of effects of cognitive maturation.

**Table 1:** **Statistical *t*-test values**.

**Contrasted age groups**	***df***	**Search RTs**			**Detection RTs**	**Cognitive latencies**
		**Target present**	**Target absent**	**Δ absent-present**		
20- vs. 18-year-olds	*t*_(24)_	<1	<1	<1	=1.0, *p* = 0.346	<1
18- vs. 16-year-olds	*t*_(24)_	<1	<1	<1	<1	<1
16- vs. 14-year-olds	*t*_(28)_	=2.5, *p* = 0.021	=1.8, *p* = 0.078	<1	<1	=2.4, *p* = 0.024
14- vs. 12-year-olds	*t*_(31)_	<1	<1	<1	<1	<1
12- vs. 10-year-olds	*t*_(32)_	=3.4, *p* = 0.002	=3.0, *p* = 0.005	=1.2, *p* = 0.239	=1.0, *p* = 0.317	=3.1, *p* = 0.004
10- vs. 8-year-olds	*t*_(29)_	=2.5, *p* = 0.018	=2.0, *p* = 0.057	<1	=3.4, *p* = 0.002	<1
8- vs. 6-year-olds	*t*_(32)_	=4.5, *p* < 0.001	=3.1, *p* = 0.004	<1	=4.8, *p* < 0.001	=2.1, *p* = 0.046

*Note: The table shows degrees of freedom (df), t-, and p-values of the t-tests comparing mean reaction times (RTs) and RT differences of target present and absent trials of the visual search task, mean RTs of the onset detection task, and mean cognitive latencies of consecutive age groups*.

**Figure 3:**
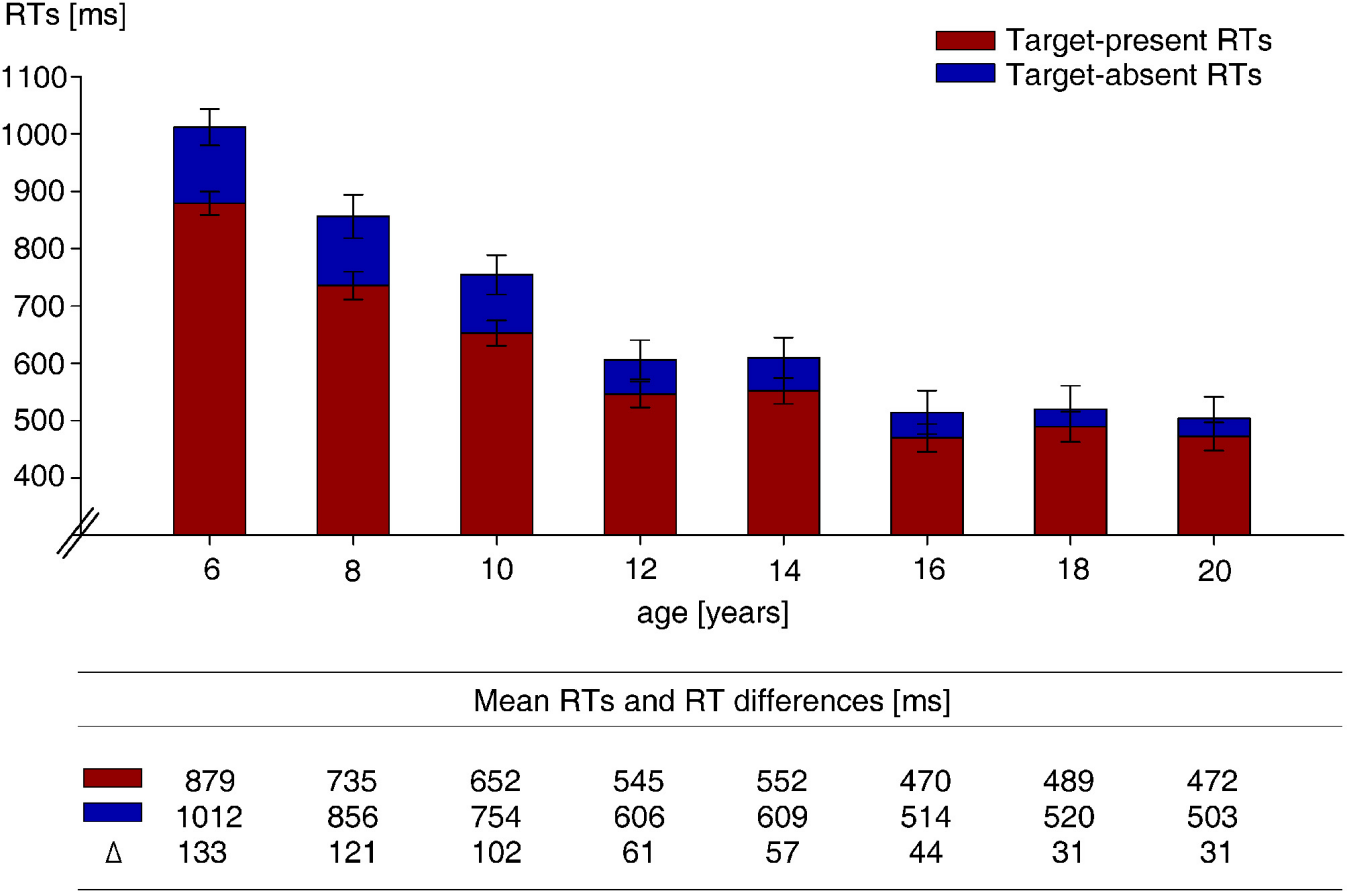
**Mean search RTs on target-absent trials of the visual search task, and mean RT differences between target-absent and -present trials**. All RTs are measured in milliseconds. Error bars reflect standard error of the mean.

Figure 3 depicts target present and absent RTs and RT differences between target present and absent trials of the visual search task separately for all age groups. A repeated measures ANOVA with the factors trial type (target absent, target present) and age revealed a main effect of trial type, *F*_(1,116)_ = 63.3, *p* < 0.001, and a significant interaction, *F*_(7,116)_ = 2.6, *p* = 0.017 between trial type and age. Participants of all age groups were faster on target present relative to absent trials, as revealed by eight separate *t*-tests to follow-up the main effect of trial type, all *t* > 2.7, all *p* < 0.016. The significant interaction between target type and age is due to a decreasing difference between target present and target absent RTs with increasing age. However, the decrease in absent vs. present RTs between successive age groups was revealed to be only numerically different, as none of the repeated contrasts was statistically significant (see Table 1). An UNIANOVA of target absent RTs of the visual search task showed a decrease with increasing age, *F*_(7,116)_ = 28.5, *p* < 0.001. Reliable differences between age groups were very similar to those on target present RTs, with (marginally) faster RTs for the 16- than the 14-year-olds, the 12- than the 10-year-olds, the 10- than the 8-year-olds (marginally), and the 8- than the 6-year-olds (see Table 1).

### Inter-trial transition effects

**Figure 4:**
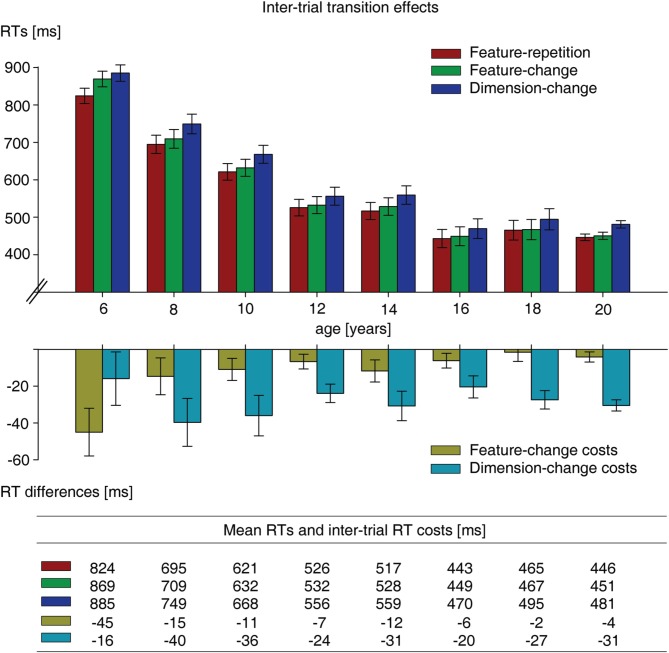
**Top panel:** Mean search RTs on feature-repetition (e.g., red target [on trial N] preceded by a red target [on trial N-1]), feature-change (e.g., red target [trial N] preceded by a blue target [trial N-1]), and dimension-change (e.g., red target [trial N] preceded by a left-tilted target [trial N-1]) trials, separately for each age group. Bottom panel: Relative feature-based (obtained by subtracting feature-change RTs from feature-repetition RTs), and dimension-based (obtained by subtracting dimension-change RTs from feature-change RTs) inter-trial RT costs, separately for each age group. All RTs are measured in milliseconds. Error bars reflect standard error of the mean.

The analysis of effects of the target definition in the previous trial on RTs in the present trial (inter-trial transition effects) involved three types of transitions with respect to the target-defining dimension and feature: dimension repetition, feature repetition (e.g., trial N-1: blue [color] → trial N: blue [color]); dimension repetition, feature change (e.g., N-1: left-tilted [orientation] → N: right-tilted [orientation]), and dimension change, feature change (e.g., N-1: red [color] → N: left-tilted [orientation]). The top panel of Figure 4 shows the mean RTs to targets of the visual search task dependent on the type of inter-trial transition, separately for each age group. RTs as a function of the three types of inter-trial transitions were subjected to a repeated-measures ANOVA with the factors inter-trial transition (feature-repetition, feature-change, dimension-change) and age. The main effect of inter-trial transition was statistically significant, *F*_(2,224)_ = 81.3, *p* < 0.001. Importantly, the transition main effect was modulated by age as indicated by a significant interaction between the two factors, *F*_(14,224)_ = 2.0, *p* = 0.019. The magnitude of the effects of target definition on the preceding trial N-1 on trial N alters as a function of age group. In order to examine the interaction, (which was predicted by the assumption of feature-based effects in children aged 6 years and dimension-based effects in adults and children above the age of 8), follow-up *t*-tests were used to statistically substantiate any such (feature-based and dimension-based) effects separately for each individual age group. Crucially, the *t*-tests revealed reliable dimension-based inter-trial effects (significantly slower RTs on dimension-change relative to feature-change trials) for participants of all age groups, all *t* > 3.4, all *p* < 0.020, with the exception of the 6-year-olds, *t*_(19)_ = 1.1, *p* = 0.568. Vice versa, significant feature-based inter-trial effects (slower RTs on feature-change relative to feature-repetition trials) were found only in the 6-year-olds, *t*_(19)_ = 3.5, *p* = 0.006, but not in the participants of any of the older age groups, all *t* < 1.9, all *p* > 0.162. The pattern of results can be seen best from the bottom panel of Figure 4 which shows the RT costs associated with feature change, and dimension change across trials, separately for each age group. Feature- and dimension-based inter-trial RT costs were subjected to UNIANOVAs in order to substantiate potential effects of age group. The UNIANOVA on dimension-based inter-trial RT effects did not reveal a significant main effect of age, *F*_(7,116)_ < 1. Dimension-based inter-trial effects did not differ across all age groups. However, in contrast to the dimension-based inter-trial effects of participants aged 8 years and older, they were not pronounced enough to reach significance in the 6-year-olds. By contrast, the main effect on feature-based inter-trial effects was statistically highly significant, *F*_(7,116)_ = 3.6, *p* = 0.002. Planned repeated contrasts showed that feature-based inter-trial effects were significantly more pronounced in the group of 6-year-olds (45 ms) compared to the 8-year-olds (15 ms), *t*_(32)_ = 2.8, *p* = 0.009. Further, comparisons of the groups of participants older than 8 years did not reveal any statistical differences in the magnitudes of feature-based inter-trial effects. The effects were the same for participants of age 8 and above, all *t* < 1.

### Errors

**Figure 5:**
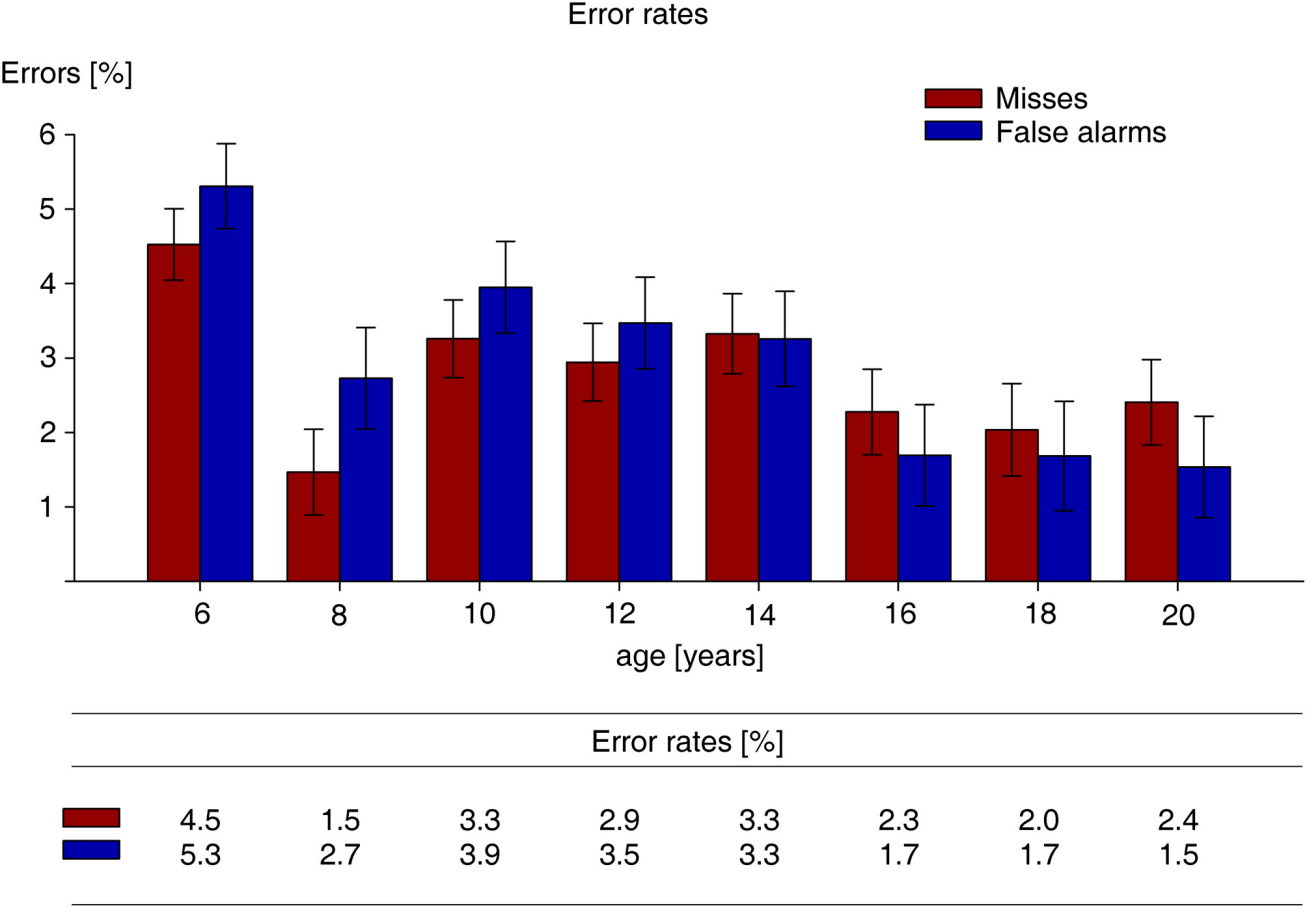
**Error rates, measured as percentage, on target-present (misses) and target-absent (false alarms) trials, separately for each age group**. Error bars reflect standard error of the mean.

Error rates on target present and absent trials of the visual search task were subjected to a repeated measures ANOVA with the factors error type (miss, false alarm) and age. The main effect of error type was not significant, *F*_(1,116)_ = 1.2, *p* = 0.273. The interaction between error type and age was statistically reliable, *F*_(7,116)_ = 2.7, *p* = 0.012. As can be seen from Figure 5, depicting miss and false alarm rates of the visual search task, this was due to the fact that in the three oldest age groups, the proportion of misses was larger than that of false alarms, while the reversed pattern was observed in the age groups of 12 years and younger. The 14-year-olds produced an equal number of misses and false alarms. Eight follow-up *t*-tests uncovered that the difference between miss and false alarm rates was only significant for the 20-year-olds, *t*_(13)_ = 2.4, *p* = 0.032, and the 8-year-olds, *t*_(13)_ = 3.4, *p* = 0.005, all other *t* < 1.8, all *p* > 0.111. Two separate UNIANOVAs on misses, *F*_(7,116)_ = 3.2, *p* = 0.004, and false alarms, *F*_(7,116)_ = 4.4, *p* < 0.001, showed a significant main effect of age. Planned repeated contrast revealed that the proportions of misses, *t*_(32)_ = 4.1, *p* < 0.001, and false alarms, *t*_(32)_ = 2.9, *p* = 0.007, were reduced in the 8- relative to the 6-year-olds, and that miss rates were also smaller for the 8- relative to the 10-year-olds, *t*_(29)_ = 2.3, *p* = 0.029; all other *t* < 1.8, all *p* > 0.105. (However, these significant differences in miss and false alarm rates between the three youngest age groups seem to reflect a special effort made by the 8-year-olds, rather than a developmental effect).

## Discussion

The main objective of the present experiment was to investigate the role of developing cognitive processes within the context of the overall search performance improvement with increasing age which has been observed in a number of studies (Trick and Enns, 1998; Gerhardstein et al., 2001; Gerhardstein and Rovee-Collier, 2002; Donnelly et al., 2007). In particular, it was proposed that the ability to abstract specific visual features into feature categories or dimensions would contribute significantly to an expedition of RTs in visual search for targets differing from distractors by a salient feature. Relying on salience representations to solve feature or pop-out search tasks (in adult observers) is characterized by a specific signature of how target definition in the preceding trial affects search RTs in the present trial: RT benefits in consecutive trials in which the target dimension is repeated and RT costs in dimension change trials. Processing target objects at the level of individual features gives rise to feature-based inter-trial effects. Accordingly, dimension-specific and feature-specific inter-trial effects were employed as a methodological tool to assess whether observers based detection of the search target on dimensional salience signals (reflecting an ability to abstract from particular features), or on featural identities (reflecting an inability to forgo processing at the feature level). The results show feature-based processing in children aged six and dimension-based processing in participants aged eight and older, suggesting that dimension-based processing of visual stimuli develops at the age between 6 and 8 years. To elaborate, the pattern of dimension-based inter-trial effects replicates previous findings in adult observers, who were shown to take the decision on target presence or absence on the basis of attentionally weighted dimensional salience signals (e.g., Müller et al., 1995; Found and Müller, 1996; Krummenacher et al., 2010). However, the 6-year-olds did not show such dimension-based RT effects over and above the benefits and costs associated with feature-based effects. Rather, they produced reliable feature-specific inter-trial effects: relative RT costs on feature change and benefits on feature repetition trials. This suggests that 6-year-olds necessarily access the level of feature representations in pop-out search tasks. This finding mirrors and extends previous results (Donnelly et al., 2007) and it fits neatly into the framework of theoretical models of cognitive development (e.g., Piaget, 1964; Frith and Frith, 1978; Tversky, 1985) proposing that the ability to categorize individual features into higher-order dimensional concepts is developed around the age of seven (if it is assumed that the ability to categorize mediates dimension-based processing). In children younger than 7 years of age processing of visual objects is based on information about the specific features characterizing an object. As hypothesized, the feature-based RT effects in the 6-year-olds reflect that feature representations activated at a given point in time (the previous trial) persist to affect processing in the immediate future (the current trial). (For previous observations of this effect on adult observers see Krummenacher et al., 2010, and Grubert et al., 2013). Finally, the absolute magnitudes of (statistically reliable) dimension-based and (statistically not reliable) feature-based inter-trial effects did not vary significantly between participants of the age groups older than eight. The magnitude of feature-based effects differed statistically reliably only between the 6- and 8-year-olds. In other words, the ability to rely search on the presence of salience signals (that is highly likely to be associated with the ability to categorize) is fully established from age eight and does not undergo further development until the age of 20 years.

Of note, theoretically it is possible that the increased feature-based inter-trial effects observed in the 6-year-olds emerged due to their overall slower mean RTs. However, in the present experiment this does not seem to be very likely for several reasons: First, if the generally slower mean RTs did cause an increase of the relative RT differences between successive trials, then not only feature-based, but also dimension-based inter-trial effects should have been increased for the 6-year-olds relative to the older age groups. Despite the fact that the dimension-based inter-trial effects of the 6-year-olds were not even statistically significant, they were also not increased compared to the dimension-based effects of the older participants (16 ms vs. 40 and 36 ms for the 8- and 10-year-olds, for example). Second, mean RTs were slowest for the 6-year-olds, but mean RTs were also reliably slower for the 8- than the 10-year-olds, the 10- than the 12-year-olds and even for the 14- relative to the 16-year-olds. Nevertheless, in no other age group feature-based inter-trial effects were statistically even close to significance, nor did they reliably differ in size for the participants aged 8 years and older. Along similar lines, the dimension-based inter-trial effects obtained in participants aged 8 years and older did not differ in size across age groups either. If, however, slower RTs were responsible for a general increase in relative inter-trial priming effects, one could expect feature- as well as dimension-based inter-trial effects to increase with decreasing age in an accelerated fashion, in accordance with mean RTs. The present data does not provide any statistical proof of such a positive relation between the overall speed of RTs and an increase in relative trial-to-trial priming, neither in a feature-based nor in a dimension-based analysis. Stated differently, the results are in line with developmental theories suggesting that 6-year-olds were not able to categorize multiple features into feature dimensions. Thus, as 6-year-olds do not possess a concept of feature dimensions, target detection is based on featural information, eliciting feature- rather than dimension-based inter-trial effects.

A second main objective of the study was to examine the development of search performance with increasing age in visual search for feature singletons. In order to do so, search RTs were related to RTs in a simple detection task that reflects signal detection and response execution (i.e., sensory-motor) processing. Based on additive factors logic, comparing search RTs and simple detection RTs allows for the isolation of effects due to cognitive development and those of sensory-motor maturation. As already observed in previous studies, search RTs decreased with increasing age (Trick and Enns, 1998; Gerhardstein et al., 2001; Gerhardstein and Rovee-Collier, 2002; Adler and Orprecio, 2006; Donnelly et al., 2007). In the present study, significant RT decreases were observed between the groups of 6- and 8-year-olds, 8- and 10-year-olds, 10- and 12-year-olds, and 14- and 16-year-olds. Overall, the decrease of search RTs as a function of age can be explained as mainly driven by sensory-motor maturation in the younger age groups up to the age of 10. By contrast, in participants of the groups of 10- and 12-, and 14- and 16-year-olds, the expedition of RTs is due to the development of search-related, that is, cognitive processes. Critically, and in contrast to the assumptions of some previous studies (Trick and Enns, 1998; Gerhardstein et al., 2001; Gerhardstein and Rovee-Collier, 2002; Adler and Orprecio, 2006), but in line with Donnelly et al. (2007), the present results show that cognitive development also occurs between the 6- and the 8-year-olds, which might (at least partially) be explained by the development of categorization skills. According to models of visual search such as the DW account (Müller et al., 1995; Found and Müller, 1996; see also Krummenacher and Müller, 2012) RTs in the age group of the 6-year olds were slower than in the other groups, because they obligatorily accessed the level of feature representations while participants of older age groups relied on the existence of a salience signal.

Further evidence for search related development was observed along the lines of Donnelly et al.'s (2007) analysis of target absent-present RT differences. In participants of all age groups RTs in target absent trials were slower than in target present trials. This is a pattern that is typically observed in feature search tasks (e.g., Chun and Wolfe, 1996). Grubert et al. (2013) argued that in target-absent compared to target-present trials observers allow for some additional time to pass before initiating a target-absent response in order for a (slow) salience peak signaling target presence to build up and reach a threshold required to elicit a target-present response. The present results show that the younger participants were, the larger was the difference between target-present and target-absent responses. Stated differently, the difference between target present and target absent RTs significantly decreased with increasing age. Numerically, the decrease in the RT difference (41 ms) was particularly large between the groups of 10- and 12-year-olds (compared with a maximum difference of 19 ms in the other comparisons between adjacent age groups). While it is difficult to conclusively account for the large decrease in the difference between present and absent RTs between the ages of 10 and 12 years, it is safe to assume that the effect is due to a cognitive difference between these two age groups. One possibility is that the younger participants approached the search task in a more conservative fashion than older participants: They tried to avoid missing the presence of a target. An interpretation along those lines is supported by the error rates: False alarm rates were higher than miss rates in all participants younger than the age of 12 years.

Overall, the pattern of RT differences between age groups of the present experiment spanning a range of 6–20 years suggests that in visual search for singleton feature targets the level of adult performance is achieved at the age of 16 years. RTs of adult observers, represented by the 20-year-olds did not differ significantly from RTs of 18-, and 16-year-olds, while in the present study RTs of 14-year-olds were significantly slower than RTs of the 16-year-olds. Importantly, the decrease in RT between ages 14 and 16 years was exclusively due to search-related or cognitive factors. The increase in search performance (expedited RTs) between 14 and 16 years may be seen as reflecting the transition between premature and adult searchers. The interpretation in terms of a transitory phase is supported by the distribution of error rates across age groups. Participants aged 16 and older produced a larger proportion of misses than false alarms, reflecting the error distribution of the 20-year-old (adult) observers. The pattern of error trials was reversed in participants younger than 14 years. Although, relative to older participants, target absent responses were initiated after a significantly longer delay relative to target present responses (see above), the false alarm rate was higher than the miss rate. The 14-year olds mark the transition with respect to the relative distribution of error types across age groups.

Taken together, children aged 8 years and older rely on a feature-less salience activity to solve visual search tasks for feature singletons. The skills required to categorize features into dimensions are acquired between the age of 6 and 8 years. It is speculated that the cognitive skills required in feature search are likely to be associated with a more general ability to categorize objects along multiple visual characteristics. The present evidence suggests that, in search for pop-out targets, the development of components mainly related to sensory-motor processing is completed by the age of 10 years. More importantly, expedited RTs due to search-related (cognitive) processes were observed between the age of six and eight (transition from feature-based to dimension-based processing), between the age of 10 and 12 (decrease in the difference between target-present and target-absent RTs), and between the age of 14 and 16 (reversal of error distributions and adult level search performance).

## Author note

The study was supported by Swiss National Science Foundation grants 100014-125205/1 to Joseph Krummenacher and PP001-110543/1 to Anna Grubert.

### Conflict of interest statement

The authors declare that the research was conducted in the absence of any commercial or financial relationships that could be construed as a potential conflict of interest.

## Footnotes

^1^The detection task was completed after the search task by all observers in order to avoid task confounds, that is, participants responding to the display onset in the search task rather the detection of the presence of an odd-one-out target item in the search display.
